# Cognitive and Emotional Impairment after Minor Stroke and Non-ST-Elevation Myocardial Infarction (NSTEMI): A Prevalence Study

**DOI:** 10.1155/2019/2527384

**Published:** 2019-04-01

**Authors:** Åse Hagen Morsund, Hanne Ellekjær, Arne Gramstad, Magnus Tallaksen Reiestad, Rune Midgard, Sigrid Botne Sando, Egil Jonsbu, Halvor Næss

**Affiliations:** ^1^Department of Neurology, Møre and Romsdal Health Trust, Molde hospital, Molde and Department of Neuromedicine and Movement Science, Norwegian University of Science and Technology, Trondheim, Norway; ^2^Department of Neuromedicine and Movement Science, Norwegian University of Science and Technology, Trondheim and Stroke Unit, Department of Internal Medicine, St Olavs hospital, University Hospital of Trondheim, Norway; ^3^Department of Neurology, Haukeland University Hospital and Department of Biological and Medical Psychology, University of Bergen, Norway; ^4^Department of psychiatry, Møre and Romsdal Health Trust, Molde hospital, Molde, Norway; ^5^Department of Neurology, Møre and Romsdal Health Trust, Molde hospital, Molde and Unit for Applied Clinical Research, Norwegian University of Science and Technology, Trondheim, Norway; ^6^Department of Neuromedicine and Movement Science, Norwegian University of Science and Technology, Trondheim and Department of neurology, St Olavs hospital, University Hospital of Trondheim, Norway; ^7^Department of Psychiatry, Møre og Romsdal Health Trust and Department of Mental Health, Norwegian University of Science and Technology, Trondheim, Norway; ^8^Department of neurology, Haukeland University Hospital, Centre for age-related medicine, Stavanger University Hospital, Institute of Clinical Medicine, University of Bergen, Norway

## Abstract

**Aim:**

To study the prevalence of cognitive and emotional impairment following a minor ischemic stroke compared to an age-matched group with non-ST-elevation myocardial infarction (NSTEMI).

**Methods:**

We included patients aged 18-70 years with a minor ischemic stroke defined as modified Rankin Scale (mRS) 0-2 at day 7 or at discharge if before and age-matched NSTEMI patients with the same functional mRS. We applied a selection of cognitive tests and the patients completed a questionnaire comprising of Hospital Anxiety and Depression scale (HADS) and Fatigue Severity Scale (FSS) at follow-up 12 months after the vascular event. Results of cognitive tests were also compared to normative data.

**Results:**

325 ischemic stroke and 144 NSTEMI patients were included. There was no significant difference in cognitive functioning between ischemic stroke and NSTEMI patients. Minor stroke patients and to a lesser extent NSTEMI patients scored worse on more complex cognitive functions including planning and implementation of activities compared to validated normative data. For the minor stroke patients the location of the ischemic lesion had no influence on the result. The prevalence of anxiety, depression, and fatigue was significantly higher in the stroke group compared to the NSTEMI group. Depression was independently associated with reduced cognitive function.

**Discussion and Conclusion:**

Minor ischemic stroke patients, and to lesser degree NSTEMI patients, had reduced cognitive function compared to normative data, especially executive functioning, on 12-month follow-up. The difference in cognitive function between stroke and NSTEMI patients was not significant. Depression was associated with low scores on cognitive tests highlighting the need to adequately address emotional sequelae when considering treatment options for cognitive disabilities.

## 1. Introduction

Acute ischemic stroke is a heterogeneous clinical syndrome and can lead to a variety of physical and cognitive clinical manifestations. Sensorimotor deficits are usually fairly evident, whereas cognitive deficits may be rather inconspicuous. However, a recent review [1] reported that poststroke dementia might affect as many as 30% of stroke patients. Fatigue has been reported in 34.7% of patients following a minor stroke [2].

Patients with minor strokes are thought to have good long-term prognosis. Sensorimotor symptoms are often marginal at admission and improve quickly the first days and weeks. Most patients are discharged directly to their homes and may have no physical neurological deficits at follow-up after few weeks. However, they may have cognitive symptoms compatible with a mild cognitive impairment or dementia [3–8] and suffer from fatigue [2, 9] and emotional symptoms [3, 10] of longer duration. Anxiety following a minor stroke is less explored [3, 11], whereas subsequent depression and fatigue after minor strokes are better known [2, 3, 10]. These symptoms may have substantial impact on daily functions, rehabilitation, and the patients' ability to stay in employment after an ischemic stroke [3, 12, 13].

High prevalence of cognitive impairment and depression after TIA and minor stroke are discussed in a systematic review from 2014 [3], but only a few of the included studies were of high quality. The studies revealed large variations in methodology and distribution of included cases [3]. The authors concluded that the knowledge of poststroke anxiety was limited. The prevalence of fatigue was high, but the studies were few and lacked relevant comparison groups. A prospective study from Turkey found significant cognitive impairment in patients with minor stroke and transient ischemic attack compared to controls [5], and, in a prospective Swiss study, poststroke fatigue was found in 34.7% at 12 months after a minor stroke [2].

Assessment of cognitive dysfunction and emotional symptoms is not routinely performed during follow-up and knowledge of the long-term impact of cognitive and emotional symptoms is sparse [3, 5], but attention to this issue is growing [13, 14]. There is no consensus on diagnostic criteria for minor stroke [15–19] which hampers research on this topic. There is consequently a need for studies investigating the consequences of cognitive and emotional impairment of minor strokes. The aim of this study was to assess the prevalence of cognitive and emotional impairment, in patients with minor ischemic stroke. Non-ST-Elevation myocardial infarction (NSTEMI) patients were selected as a control-group because both conditions implicate symptomatic vascular disease and related risk factors with the intracerebral lesion as the difference. Comparison with normative data was included for the cognitive tests.

## 2. Material and Methods

A 12-month follow-up was performed with a selection of cognitive tests and a questionnaire on anxiety, depression, and fatigue. The presence of anxiety and/or depression is defined as emotional impairment. A control group of NSTEMI patients was chosen.

Emotional symptoms in our study is defined as anxiety and depressive symptoms and fatigue.

*Patients* were as follows*: *ischemic stroke was defined in accordance to the Baltimore-Washington Cooperative Young Stroke Study Criteria [20] comprising neurological deficits lasting more than 24 hours because of ischemic lesions or transient ischemic attacks where CT or MRI showed infarctions related to the clinical findings.* Inclusion criteria* were as follows: ischemic stroke patients 18-70 years with minor ischemic stroke defined as mRS 0-2 [21] at day 7 or at discharge if before and TIA-patients with an ischemic lesion verified at MRI. NSTEMI patients aged 18-70 years with mRS 0-2.

*Exclusion criteria *were as follows: patients with a major stroke defined as mRS > 2 day 7 or at discharge if before and patients with deterioration in mRS to more than 2 in the observational period of any cause. NSTEMI patients with mRS > 2 of any cause.

*Recruitment* was as follows; ischemic stroke patients were recruited consecutively from stroke units at Molde Hospital, Haukeland University Hospital, and St Olav's Hospital. Patients from other participating stroke units, Department of Neurology at St Olavs Hospital, Kristiansund Hospital, Volda Hospital, and Aalesund Hospital, were included whenever practical, but not always consecutively. Recruitment period was 01/01/13-12/31/16. NSTEMI patients were recruited from Haukeland University Hospital, Ålesund Hospital, Molde Hospital, and Kristiansund Hospital in the same time.

*Baseline investigation* was as follows: ischemic stroke patients underwent routine examination with NIHSS [22], traditional risk factors including hypertension, diabetes mellitus, hypercholesterolemia, smoking, BMI ≥ 25, and brain imaging with CT and/or MRI. Patients were treated according to Norwegian guidelines for ischemic stroke. Most patients with mRS ≤ 2 after 7 days or at discharge had no need for further rehabilitation and were discharged to their home.

Demographic data were collected from the initial admission time for the ischemic stroke and NSTEMI.

## 3. Assessment of Cognitive and Emotional Function

Clock drawing test [23] were used as screening of global cognitive function. Clock drawing test assesses visuospatial function [23]. Trail-making tests A and B, Color-Word interference test, and Verbal fluency (FAS) were used to test executive function [24].The Color-Word interference test is divided into 4 items: color naming, color reading, inhibition and inhibition/switching, testing mental flexibility, mental speed, and inhibition. These tests were drawn from the Delis–Kaplan Executive Function System (D-KEFS). D-KEFS was developed to provide reliable normative data for a range of executive functions [25].

Memory was tested with the CERAD ten-words learning task [26]. CERAD (Consortium to Establish a Registry for Alzheimer's Disease) is standardized validated test battery for the assessment of Alzheimer disease [26] with normative data adjusted for age and education [27]. Minor stroke and NSTEMI patients were compared with validated normative data for Trail-making test A and B, Color-Word interference test, Verbal fluency, and Ten-word learning task [26, 28].

Scores falling below 1.5 SD of the mean were characterized as abnormal.

Ischemic stroke patients were screened by the Ullevål aphasia screening test [29].

### 3.1. Questionnaires

Hospital Anxiety and Depression scale (HADS) was used to assess anxiety and depression [30]. A score ≥ 8 on the anxiety (HADS-A) or depression (HADS-D) items indicates possible presence of anxiety or depression disorders [30]; a total score ≥ 15 indicates a mixture of anxiety and depression.

Fatigue Severity scale (FSS) was used to assess fatigue [31]. FSS is a nine-item questionnaire that assesses the effect of fatigue on daily living. Each item is a statement on fatigue that the subject rates from 1, “completely disagree”, to 7, “completely agree”. [32] Fatigue is defined as FSS score ≥ 5 [32].

Cognitive testing was performed by trained research nurses or by the neurologist responsible for the study.

Written informed consent was obtained from the patients for their anonymized information to be published in this article.

The ethics committee of Rogaland, Hordaland, and Sogn and Fjordane (REC west) approved this study (REC no. 2012/1708).

## 4. Statistics

Student's* t*-test was used to assess differences in mean values. Chi square was used to assess differences in categoric variables. Multivariable linear regressions were used to assess association between more than two variables. The level og significans was set to p=.05. All significance testing was done as two-tailed tests.

STATA 14 (Statacorp 4905 Lakeway Drive, College Station, Texas 77845 USA) was used for analyses.

## 5. Results

In total, 324 ischemic stroke patients were included at admission and followed up at 3 months, and 288 were followed up at 12 months. Either a phone call or a letter recruited the NSTEMI group after their vascular event. 144 of 146 NSTEMI patients who accepted participation completed the questionnaire and cognitive tests.  Table 1 shows baseline demographic data on ischemic stroke and NSTEMI patients. The mean age for ischemic stroke patients was significantly lower than for the NSTEMI group, 58 years (SD = 10.0) and 60 years (SD = 6.6), respectively. Proportion of women was significantly higher in the ischemic stroke group. There were significantly more NSTEMI patients who smoked compared to the ischemic stroke patients (p =.04). More NSTEMI patients than ischemic stroke patients were employed (p =.03). The mean value for premorbid mRS in ischemic stroke patients was 0.3 (SD = 0.6), mRS at discharge/day 7 was 1.2 (SD= 0.8), and NIHSS at discharge/day 7 was 0.8 (SD = 1.0). The number of patients treated with antidepressant was 24 (8%) and in the control group 10 (7%).

### 5.1. Cognitive Function

Table 2 shows results of the cognitive tests, HADS, and FSS in ischemic stroke and NSTEMI patients. Cognitive tests were compared with each other and normative data for both groups.

Clock-drawing test score was 5 in both groups, indicating no deficit in either group on this test.

There were no differences between ischemic stroke and NSTEMI patients in Ten-word learning task, Trail-making tests A or B, Verbal fluency, or Color-Word Interference tests except for the subtest Inhibition/Switching (p =.03) where the stroke group scored worse.

Ischemic stroke patients scored worse than normative data for Trail making test B (p =.02), Color naming (p <.001), Color reading (p =.001), Inhibition (p <.001), and Inhibition/Switching (p =.000). NSTEMI patients scored worse than normative data in the subtest Color naming (p =.02) and Inhibition/Switching (p =.02).

The percentage of subjects with ≥2 abnormal cognitive tests was 77% in the ischemic stroke patients, and 84% in the NSTEMI patients (p=.05).

There was a significant gender difference in the color-word inhibition test in the patient group where males soored worse (p=.05), but no significant gender difference in the control group (table available on request).

Table 3 shows that performance on most tests (memory, mental flexibility, mental speed, and inhibition and executive functions) was independently associated with education and depression and, to a lower degree, with age, gender, and fatigue. Stratifying age groups did not change this (table available on request). We performed the same analyses in the stroke and NSTEMI patients separately and found that the association between age and cognitive impairment became significant in one more test (5 of 13 cognitive tests), wheras the result for the NSTEMI group was unchanged. There was an association between diabetes mellitus and color inhibition and between overweight and inhibition error (table ii in suppmelentary data).

There were significant differences as a function of laterality in Verbal fluency and the inhibition part of the Color-Word inhibition test, with higher prevalence of deficient scores if infarctions were located in the left versus right hemisphere (Table 4). There were no significant differences in cognitive impairment, anxiety, depression, and fatigue between subcortical and cortical infarctions (Table 4).

The mean value of the aphasia score at 12 months in the ischemic stroke patients was 51.7 (SD = 1.1) of a total of 52 points.

*Fatigue* was as follows: the prevalence of fatigue was higher in the ischemic stroke than NSTEMI patients (29% versus 15%, p =.002).

### 5.2. Anxiety and Depression

There was a difference in the prevalence of combined anxiety and depression measured by total HADS score between ischemic stroke and NSTEMI patients, with a significantly larger proportion of stroke patients (16%) scoring above the cutoff than NSTEMI patients (0%) (p =.00). However, there were no significant differences in the symptoms of either anxiety (p =.2) or depression (p =.06).

## 6. Discussion

### 6.1. Cognitive Function

The differences between patients with ischemic stroke and NSTEMI were not statistically significant on most tests, even though the ischemic stroke patients scored numerically worse on all cognitive tests except verbal fluency and reading errors. This trend may suggest that the ischemic stroke patients have slightly more encompassing cognitive problems than NSTEMI patients do. However, given that ischemic stroke patients have demonstrated brain lesions, it is surprising that the difference on cognitive function between the two groups is so slight. One explanation may be the low NIHSS. We only found a modest association between the risk factors diabetes and overweigt, and impairment of the cognitive tests as shown in table ii in supplemental material. Cerebrovascular risk factors are associated with development of cerebrovascular changes, which increases with increasing age. An age-dependent association between smoking and white matter hyperintensities is found [33]. Our patients population is relatively young, something which may explain the lack of association between cognitive impairment and risk factors. It is known that cerebrovascular risk factors also increases the risk of Alzheimer's disease [34] and our findings may reflect a preclinical stage of this neurodegenerative disease. NSTEMI patients may also have vascular changes in the brain; this is unknown because no brain imaging was done in the NSTEMI group.

The NSTEMI group scored significantly worse on some cognitive tests compared to the normative data (Table 2), which suggests that NSTEMI patients also have a cerebrovascular disease. This finding is consistent with a prior study that found that cognitive outcomes after a coronary syndrome were similar to minor stroke [35]. Both ischemic stroke and NSTEMI patients may have prior cerebrovascular lesions, but our study has no data on this. We therefore do not know if prior cerebrovascular lesions can explain this lack of difference. Furthermore, many NSTEMI patients are treated with a coronary intervention, which can cause silent infarctions in the brain, even though a recent study did not find significant cognitive impairment after coronary angiography and percutaneous coronary intervention [36, 37]. However, NSTEMI patients in our study are significantly older compared to the minor stroke patients which increases the risk of neurodegenerative diseases such as early Alzheimer's disease.

The proportion with more than two abnormal cognitive tests was 77% in ischemic stroke patients and 84% in NSTEMI patients. This suggests that both patient groups are associated with a generalized cognitive dysfunction. It also highlights that there are important cognitive sequelae following vascular disease for the majority of the patients regardless of where the disease manifests itself.

Patients with ischemic stroke showed significant deficits in several cognitive domains compared to normative data. Areas of dysfunction include mental flexibility, mental speed, and inhibition. These higher order cognitive functions underpin executive functioning, which encompass supervisory skills that are crucial to successful and purposeful goal-directed behavior [38]. These functions are important in planning and implementation of activities. Impaired executive dysfunction can have detrimental effect on social function and the ability to stay in work.

Increasing age showed significant correlation with lower scores on several cognitive tests, as expected because cerebrovascular changes increase with increasing age. The slight difference in the results of the cognitive tests in the stroke and NSTEMI group is hardly clinical significant. Males showed significantly lower scores on several cognitive tests than females at the same age, despite no difference in educational level, which can possibly be explained by higher load of vascular disease in males [39]. This effect was clearer in the NSTEMI patients.

Employment was more frequent among NSTEMI patients than among ischemic stroke patients even though NSTEMI patients were older. A possible explanation is that some ischemic stroke patients may have had cognitive disabilities prior to the index stroke because of chronic cerebrovascular disease. Perhaps a more insidious onset of cognitive difficulties in the NSTEMI patients permits a gradual accommodation to their vocation despite cognitive difficulties.

The location of the ischemic lesion only influenced two of 13 cognitive tests and did not seem to be a strong determining factor for cognitive function. However, both tests were verbal and were more affected by left hemisphere lesions, which is in the direction expected given that language normally is located in the left hemisphere.

### 6.2. Fatigue

We found a high prevalence of fatigue among the ischemic stroke patients, which is consistent with prior studies [2, 3, 9, 40–42]. The prevalence in other studies varies between 23 and 40%, compared to 29% in our study. The prevalence in the NSTEMI-group was low compared to the study of Eckhardt et al. [43]. Younger NSTEMI patients and fewer patients with cardiac failure in our study may partly explain these discrepancies.

### 6.3. Anxiety and Depression

The prevalence of combined anxiety and depression (HADS>15) was significantly higher in the ischemic stroke patients than NSTEMI patients, but the difference in pure anxiety or depression symptoms was not significant. The scores were compared to the prevalence of anxiety and depression in the population-based study of HUNT (The Nord-Trondelag Health study) [44] where about 9% of the population reported elevated anxiety and 5% elevated depression (on HADS). There were significantly more anxiety and depression disorders in the ischemic stroke group than in the study of HUNT. However, this was not the case for the NSTEMI group.

As expected, reported depression was associated with lower scores on some cognitive tests. Cognitive dysfunction among patients with reported depression is a common finding [45]. Reported anxiety was not associated with change in cognitive performance. This finding is in contrast to prior studies investigating a stroke population [46] and in healthy individuals [47]. Both of these studies found that anxiety had a detrimental impact on cognitive functions. Anxiety and depression disorders are treatable and may hamper recovery if untreated. Depression is in itself found to be associated with worsening and enduring cognitive dysfunction even after remission [48]. The current findings of a broad cognitive dysfunction in both patient groups suggest that they may have less cognitive reserves and may therefore be extra vulnerable to events of adverse effects on cognitive function including depression.

*Strengths and weaknesses* are as follows: strength of this study is the large number of patients and inclusion of controls. It is a multicenter study and reflects the patients in the middle and western part of Norway. The existence of normative data made it possible to detect a significant difference for both patients and controls compared to the normative data.

There are some weaknesses. Recruitment of individuals to the control group was difficult which may have biased selection. Different study nurses and one neurologist performed testing. Some patients live far from the hospital. A long traveling distance may have influenced the ability to concentrate in performing cognitive tests. Lack of registration of proportion of percutaneous coronary intervention (PCI) in the NSTEMI-group is another limitation, because PCI may cause ischemic lesions in the brain as shown in a study from 2016 [36, 37].

## 7. Conclusion

We found no significant difference in cognitive function between minor ischemic stroke patients and NSTEMI patients. However, the stroke patients and to a lesser degree NSTEMI patients had reduced cognitive function compared to normative data, especially executive function, on long term follow-up. Prevalence of cognitive impairment increased with increasing age. Presence of risk factors and gender seemed not to substantially influence cognitive function. Depression was independently associated with low scores on cognitive tests, suggesting that treatments targeting depression can be a valuable approach to improve cognitive disabilities in these patients. The prevalence of anxiety, depression, and fatigue was significantly higher in the stroke group compared to the NSTEMI group.

## Figures and Tables

**Table 1:** Characteristics of patients and controls at baseline (time for event).

	Ischemic stroke n 324 (%)	NSTEMI n 144 (%)	p value
Age (mean)	58.0 (10.0*∗*)	60.3 (6.6*∗*)	.009
Females	120 (37)	31 (22)	.001
Education (1,2,3)^a^	51 (16)	20 (14)	.07
	148 (46)	68 (47)	
	121 (37)	56 (39)	
Employed before event	217 (67)	111 (77)	.03
Risk factors			
Hypertension	174 (54)	68 (47)	.2
Diabetes mellitus	39 (12)	22 (15)	.3
Atrial fibrillation	46 (14)	15 (10)	.3
Hypercholesterolemia^b^	151 (47)	80 (56)	.07
Smoking^c^	114 (35)	65 (45)	.04
BMI mean	26.7 (4.4*∗*)	27.4 (3.7*∗*)	.06
Overweight (BMI≥25)	189 (58)	98 (68)	<.001

^a^1 ≤ 10 years of education, 2 10-13 years of education, ≥ 14 years of education, ^b^hypercholesterolemia is defined as treatment with cholesterol lowering medication, and ^c^smoking is defined as current smoker or smoking within the last 12 months. *∗*SD.

**Table 2:** Chi square test to compare cognitive tests HAD and FSS among minor ischemic strokes and NSTEMI patients. Proportion of patients and controls with abnormal results based on 5 percentile for normative data. Significant difference with normative data is labeled with*∗*.

	Ischemic stroke N=288(%)	NSTEMI N=144 (%)	P-value^h^
10-words learning task^a^	17(6)	9(6)	1.0
10-words learning task, delayed recall^ab^	27(10)	12(8)	.7
Trail-making A^c^	15(5)	4(3)	.2
Trail-making B^c^	39(14)*∗*	16(11)	.4
Verbal fluency^c^	32(11)	17(12)	.8
*Color-Word Interference tests*			
Color naming^c^	59(20)*∗*	20(14)	.09
Color reading^c^	52(18)*∗*	17(12)	.07
Color inhibition^c^	58(20)*∗*	20(14)*∗*	.1*∗*
Color inhibition/switching^c^	58(20)*∗*	17(12)	.03
Error scores			
Naming errors^d^	16(6)	5(3)	.8
Reading errors^d^	29(10)	17(12)	.6
Inhibition errors^c^	18(6)	6(4)	.4
Inhibition/switching errors^cc^	25(9)	8(6)	.1
Proportion with ≥2 abnormal cognitive tests	220(77)	122(84)	.05
*Questionnaire*			
HADS ^e^	45(16)	0(0)	<.001
HADS-A^f^	52(19)*∗*	19(13)	.2
HADS-D ^f^	34(9)*∗*	9 (6)	.06
FSS (n=279/142) ^g^	81(29)	22(15)	.002

^a^Adjusted for age and educational level, ^b^tested with 5 minutes delay, ^c^scaled score, and ^d^cumulative percentage. ^5^Anxiety and/or depression was defined as HADS ≥ 15, ^f^anxiety was defined as HADS-A≥8, and ^f^depression was defined as HADS-D≥8. Fatigue defined as FSS ≥ 5. ^h^p value between ischemic stroke and NSTEMI *∗*P value ≤0.05 compared with normative data.

**Table 3:** Linear regression analyses with cognitive tests as dependent variables showing beta values for all independent variables (beta).

	Age	Males	Ischemic stroke vs NSTEMI	Higher education	Marital stage	HAD-A	HAD-D	FSS
10-words learning task^a^	-.17*∗*	-.18*∗*	.19*∗*	.15*∗*	-.04	-.08	-.03	-.06
10-words learning task- delayed^ab^	-.20*∗*	-.17*∗*	.04	.13*∗*	-.05	-.07	-.03	-.13
Trail-making A^c^	-.04	-.002	-.04	.26*∗*	-.09	.08	-.09	.02
Trail-making B^c^	-.04	-.08	-.03	.33*∗*	-.04	-.03	-.13*∗*	-.02
Verbal fluency^c^	-.09*∗*	-.10*∗*	.01	.30*∗*	-.01	.01	-.08	-.10
*Color-word interference tests*								
Color naming^c^	.01	-.12	-.06	.19*∗*	-.06	.03	-.19	-.08
Color reading^c^	.08	-.003	-.07	.24*∗*	-.02	.12	-.15*∗*	-.10
Color inhibition^c^	-.02	-.10*∗*	-.08	.27*∗*	-.002	.02	-.09	-.13*∗*
Color inhibition/switching^c^	-.02	-.09*∗*	-.06	.24*∗*	.01	.03	-.09	-.15*∗*
Error scores								
Naming errors^d^	-.11*∗*	-.02	-.04	.03	.06	-.02	-.17*∗*	.06
Reading errors^d^	-.04	-.11	.04	-.04	.14	.07	.06	-.10
Inhibition errors^c^	-.09	.06	-.06	-.01	-.04	.06	-.13	-.05
Inhibition/switching errors^c^	.06	-.06	-.07	.16*∗*	.01	.03	.00	-.13*∗*

^a^Not adjusted for age and educational level in a linear model, ^b^tested with 5 minutes delay, ^c^scaled score adjusted for age, and ^d^cumulative adjusted for age percentage. Significant difference with normative data is labeled with *∗*.

**Table 4:** Chi square analyses of correlation between cognitive and emotional symptoms and location of infarction.

	Subcortical infarctions n= 108 (%)	Cortical infarctions n=97 (%)	p	Left side n= 94 (%)	Right side n= 85 (%)	p
10-word learning task^a^	10 (9.4)	3 (3.1)	.07	7 (7.5)	4 (4.6)	.4
10-word learning task delayed^ab^	10 (9.4)	6 (6.3)	.4	7 (7.6)	7 (8.2)	.9
Trail-making A^c^	6 (5.6)	5 (5.2)	.9	5 (5.3)	6 (6.9)	.7
Trail-making B^c^	19 (17.6)	12 (12.4)	.3	17 (18.1)	8 (9.2)	.08
Verbal fluency^c^	15 (13.9)	15 (15.5)	.8	17 (18.1)	7 (8.1)	.05
*Color-Word Interference tests*						
Color naming^c^	23 (21.3)	21 (21.7)	1.0	18 (19.2)	14 (16.1)	.6
Color reading^c^	24 (22.4)	15 (15.5)	.2	21 (22.3)	11 (12.6)	.09
Color inhibition^c^	16 (14.8)	15 (15.6)	.9	21 (22.3)	5 (5.6)	.001
Color inhibition/switching^c^	24 (22.2)	22 (22.3)	.9	21 (22.3)	15 (17.2)	.4
Error scores						
Naming errors^d^	6 (5.6)	6 (6.2)	.8	8 (8.5)	5 (5.6)	.5
Reading errors^d^	9 (8.3)	10 (10.3)	.6	8 (8.5)	10 (11.5)	.5
Inhibition errors^c^	8 (7.4)	4 (4.2)	.3	11 (11.8)	5 (5.8)	.2
Inhibition/switching errors^c^	6 (5.6)	10 (10.4)	.2	8 (8.6)	9 (10.3)	.7
No of abnormal cogn tets	40 (38.8)	32 (33.3)	.4	36 (39.6)	24 (28.2)	.1
*Questionnaires*						
HADS^e^	23 (22.6)	11 (12.1)	.06	18 (19.8)	11 (13.4)	.3
HADS-A ^f^	25 (24.3)	15 (16.1)	.2	22 (23.9)	12 (14.5)	.1
HADS-D ^f^	13 (12.4)	12 (12.9)	.9	11 (12.0)	11 (12.9)	.8
Fatigue severity scale (FSS) ^g^	32 (30.2)	24 (25.5)	.5	26 (27.7)	23 (28.1)	1.0

^a^Adjusted for age and educational level, ^b^tested with 5 minutes delay, ^c^scaled score, and ^d^cumulative percentage.^e^Anxiety and/or depression was defined as HADS ≥ 15, ^f^anxiety was defined as HADS-A≥8, and ^f^depression was defined as HADS-D≥8. Fatigue was defined as FSS ≥ 5.

## Data Availability

The data used to support the findings of this study are available from the corresponding author upon request.

## Supplementary Materials

Supplementary Materials Table i. Chi square test of gender differences in risk factors*∗*, cognitive tests, HADS, and FSS in minor stroke and NSTEMI patients. Table ii. Linear regression analyses with cognitive tests as dependent variables showing beta values for all independent variables (beta).
