# Supplementary material for: Cognitive and Emotional Impairment after Minor Stroke and Non-ST-Elevation Myocardial Infarction (NSTEMI): A Prevalence Study
Source: Stroke Res Treat. 2019 Apr 1;2019:2527384. doi: 10.1155/2019/2527384 (PMC6463585; doi:10.1155/2019/2527384)
Supplement: Supplementary Materials — Table i. Chi square test of gender differences in risk factors∗, cognitive tests, HADS, and FSS in minor stroke and NSTEMI patients. Table ii. Linear regression analyses with cognitive tests as dependent variables showing beta values for all independent variables (beta). [file 2527384.f1.pdf]

Cognitive and emotional impairment after minor stroke and non-ST-elevation myocardial infarction (NSTEMI). A prevalence study.

Supplementary material

Table i. Chi square test of gender differences in risk factors*, cognitive tests, HADS and FSS in minor stroke and NSTEMI patients

	Male pat (%) n=186	*Female pat (%) n=102*	*p*	*Male cont (%) n= 113*	*Female cont (%) n=31*	*p*
Hypertension[a]	114 (56)	59 (49)	.2	58 (51)	11 (35)	.002
Diabetes mellitus	23 (11)	16 (13)	.6	16 (14)	5 (16)	.8
Overweight (BMI≥25)	129 (63)	60 (50)	.02	81 (72)	16 (52)	.04
Atrial fibrillation	33 (16)	13 (11)	.2	13 (12)	2 (6)	.4
Hypercholesteroaemia	98 (48)	53 (44)	.5	64 (57)	16 (52)	.6
Smoking[b]	72 (35)	42 (35)	1	51 (50)	14 (45)	1
10-words learning task[c]	12 (6)	5 (5)	.6	7 (6)	2 (6)	1
10-words learning task delayed[cd]	18 (10)	4 (4)	.08	10 (9)	2 (6)	.7
Verbal Fluency[e]	25 (13)	7 (7)	.09	14 (12)	3 (10)	.7
Trail-making A[e]	10 (5)	5 (5)	.9	2 (2)	2 (7)	.1
Trail-making B[e]	22 (12)	11 (11)	.8	13 (12)	3 (10)	.8
Color-Word Interference tests						
Color-word naming[e]	39 (21)	18 (18)	.5	16 (14)	4 (13)	.9
Color-word reading[e]	32 (17)	20 (20)	.6	14 (12)	3 (10)	.7
Color-word inhibition[e]	30 (16)	8 (8)	.05	17 (15)	3 (10)	.4
Color-word inhib/switch[e]	43 (23)	15 (15)	.1	1 (1)	1 (3)	.3
Error scores						
Color naming error[f]	10 (5)	5 (5)	.9	5 (4)	0 (0)	.2
Reading error[f]	22 (12)	7 (7)	.2	15 (13)	2 (6)	.3
Inhibition error[e]	9 (5)	8 (8)	.3	4 (4)	2 (6)	.5
Inhibition/switching error[e]	18 (10)	7 (7)	.4	7 (6)	1 (3)	.5
Impairment ≥2 cogn tests	69 (38)	29 (29)	.2	94 (83)	28 (90)	.3
Questionnaires						
HADS[g]	31 (18)	14 (14)	.4	0	0	

HADS-A[h]	33 (19)	19 (19)	1.0	12 (11)	7 (24)	.06
HADS-D[h]	26 (15)	8 (8)	.09	7 (6)	2 (7)	.9
FSS[i]	52 (29)	30 (31)	.8	14 (13)	7 (24)	.1

*Prevalence at baseline, no of male patients 204, female patients 120.[a] Hypercholesterolemia is defined as treatment with cholesterol lowering medication, [b]Smoking is defined as current smoker or smoking within the last 12 months.[c] Adjusted for age and educational level,[d] tested with 5 minutes delay,[e] scaled score, [f]cumulative percentage. [g]Anxiety and/or depression defined as HADS ≥ 15, [f]anxiety was defined as HADS-A≥8, and [f]depression as HADS-D≥8. [i]Fatigue defined as FSS ≥ 5.
Missing values in some of variables. No of female patients 97-102 male patients 172-186, no of female controls 28-31 male controls 109-113.

Table ii. Linear regression analyses with cognitive tests as dependent variables showing beta values for all independent variables (beta).

	Age	*Males*	*Ischemic stroke vs NSTEMI*	*Higher edu-cation*	*Marital stage*	*Hypertension*	*Diabetes mellitus*	*Overweight*
10-words learning task[a]	-.1	-.15*	-.04	.16*	.01	-.09	.01	-.004
10-words learning task-delayed[b]	-.16*	-.12*	-.28*	.12*	-.01	-.02	-.04	-.02
Trail-making A[c]	-.03	.02	-.08	.33*	-.03	-.05	-.06	-.03
Trail-making B[c]	-.01	-.04	-.12	.33*	-.04	-.1	-.03	-.05
Verbal fluency[c]	-.12*	-.05	.04	.29*	.02	.11	-.06	.15
Color-word interference tests								
Color naming[c]	.07	-.1	-.14	.21*	-.03	.02	-.02	-.03
Color reading[c]	.1	.01	-.11	.27*	-.004	-.03	-.02	.01
Color inhibition[c]	.02	-.08	-.11	.28*	.04	.04	-.12*	.05
Color inhibition/switching[c]	.03	-.07	-.11	.29*	.04	.04	-.1	.02
Error scores[d]								
Naming errors[d]	-.14*	.01	-.04	.02	.09	.02	.04	.06
Reading errors	-.03	-.12*	.02	.04	-.15*	.02	.07	.09
Inhibition errors[c]	-.12	.07	.05	-.05	-.05	-.7	-.03	.22*
Inhibition/switching errors[c]	.08	-.06	.02	.15*	.03	.07	-.07	.11
Number of cogn test impairment	.02	.05	.12	-.25*	.06	.05	.05	-.01

	Atrial fibrilation	*Hyper-cholesterolaemia*	*Smoking*	*HADS*	*HAD-A*	*HAD-D*	*FSS*
10-words learning task[a]	.02	-.08	-.02	-.15-.	.1	-.07	-.05
10-words learning task-delayed[b]	.08	-.09	-.04	.04	.04	-.11	-.09
Trail-making A[c]	.06	.07	-.04	.07	.02	-.07	-.06
Trail-making B[c]	.03	.04	-.02	-.18	.07	-.06	.02

Verbal fluency[c]	.01	-.04	-.06	-.1	.08	-.05	-.06
Color-word interference tests							
Color naming[c]	-.04	-.04	-.01	.07	.01	-.27*	-.03
Color reading[c]	.03	-.02	.02	.03	.05	-.12	-.04
Color inhibition[c]	-.02	-.03	-.04	.12	-.04	-.22*	-.03
Color inhibition/switching[c]	-.02	-.04	-.03	.12	-.03*	-.23	-.04
Error scores[d]							
Naming errors[d]	.08	-.06	-.01	.13	-.05	-.2*	.06
Reading errors	.09	-.08	.08	.05	-.03	.01	-.04
Inhibition errors[c]	.02	.1	.04	-.07	.07	-.08	-.03
Inhibition/switching errors[c]	-.07	-.01	-.07	-.03	.09	-.07	-.06
Number of cogn test impairment	-.01	.05	-.02	.1	-.17*	.26*	.01

[a]Not adjusted for age and educational level in a linear model, [b]tested with 5 minutes delay, [c]scaled score adjusted for age, [d]cumulative adjusted for age percentage. Significant difference with normative data is labeled with*.
